# Platelet-Derived Growth Factor Over-Expression in Retinal Progenitors Results in Abnormal Retinal Vessel Formation

**DOI:** 10.1371/journal.pone.0042488

**Published:** 2012-08-03

**Authors:** Per-Henrik D. Edqvist, Mia Niklasson, Manuel Vidal-Sanz, Finn Hallböök, Karin Forsberg-Nilsson

**Affiliations:** 1 Department of Immunology, Genetics and Pathology and Science for Life Laboratory, Uppsala University, Uppsala, Sweden; 2 Department of Ophthalmology, University of Murcia, Murcia, Spain; 3 Department of Neuroscience, Uppsala University, Uppsala, Sweden; Children's Hospital Boston, United States of America

## Abstract

Platelet-derived growth factor (PDGF) plays an important role in development of the central nervous system, including the retina. Excessive PDGF signaling is associated with proliferative retinal disorders. We reported previously that transgenic mice in which PDGF-B was over-expressed under control of the nestin enhancer, nes/tk-PdgfB-lacZ, exhibited enhanced apoptosis in the developing corpus striatum. These animals display enlarged lateral ventricles after birth as well as behavioral aberrations as adults. Here, we report that in contrast to the relatively mild central nervous system phenotype, development of the retina is severely disturbed in nes/tk-PdgfB-lacZ mice.

In transgenic retinas all nuclear layers were disorganized and photoreceptor segments failed to develop properly. Since astrocyte precursor cells did not populate the retina, retinal vascular progenitors could not form a network of vessels. With time, randomly distributed vessels resembling capillaries formed, but there were no large trunk vessels and the intraocular pressure was reduced. In addition, we observed a delayed regression of the hyaloid vasculature. The prolonged presence of this structure may contribute to the other abnormalities observed in the retina, including the defective lamination.

## Introduction

Formation of blood vessels in the mammalian eye involves extensive tissue reorganization including regression of embryonic vascular structures. The developing murine eye is initially supplied with oxygen and nutrients by the hyaloid vasculature (HV), which is later replaced by the retinal vasculature [1]. The HV is formed in the primitive vitreous body between embryonic days (E) 10.5 and E13.5. Concomitant with the postnatal (P) formation and maturation of the intraretinal vasculature, the HV degenerates via apoptosis, beginning on P4 and culminating on P7–8. On P10 most of the HV vessels have regressed and although complete regression of the hyaloid takes a few weeks, the vitreous body is completely avascular by P16 [2]. Vascularization of the retina is preceded by colonization with Pax2-positive astrocyte precursors that form a network, which becomes covered by endothelial cells [3], [4]. As they differentiate, these precursor cells begin to express GFAP as well, and change their morphology [5].

Failure of the HV to regress, results in a congenital condition known as Persistent Fetal Vasculature Syndrome (PFVS), or persistent hyaloid vasculature (PHV) [6]. The consequences can be severe intraocular hemorrhage, cataract and retinal detachment due to forces exerted on the neural retina by contractile cells associated with the abnormal vessels in the vitreous [1]. Although transgenic mouse models have shed some light on possible pathways the precise molecular and cellular mechanisms underlying the failure of the HV to regress are not yet fully understood. Disturbance of hyaloid vessel regression was reported in mice deficient in both Wnt7b-dependent and Wnt7b-independent Fzd4 signaling [7] and formation of the deeper plexus is also disrupted in these mutant mice. Wnt7b is believed to be produced by the macrophages that play key roles in the regression of capillaries of the HV [8] as indicated by the finding that in heterozygous BMP4 +/−, which lack macrophages in the vitreous, the HV persists [9]. Moreover, Arf knockout mice [10] and certain p53-null strains [11], both of which proteins are tumor suppressors, also display persistent HV, as do Ang-2 knockout mice [12].

Platelet-derived growth factor (PDGF) is essential for proper development of the retina and has been associated with proliferative retinopathies [13]. The PDGF family consists of four ligands, designated A, B, C and D that function as homodimers or in the case of AB, also as a heterodimer. PDGF-AA, -AB, -BB and -CC activate the PDGF receptor-α (PDGFRα) while PDGF-BB and –DD bind to PDGFRβ. In the normal eye, PDGF-A is expressed by both neurons and astrocytes [14] and, together with PDGFRα, regulates the recruitment of astrocyte precursors to the retina and their subsequent development at this location [14], [15]. In this manner, interactions between PDGF-A and PDGFRα determine the number and distribution of astrocytes in the retina. Maintenance of the retinal vasculature depends on signaling by PDGF-B via the PDGFRβ. Pericytes express PDGFRβ [16], [17] and their attachment to vessels is dependent on PDGF released from endothelial cells.

Transgenic over-expression of PDGF-A in retinal ganglion cells (RGCs) results in a dose-dependent increase in the proliferation of GFAP-immunoreactive (+) cells in the retina, as well as inhibiting the migration and spread of these cells across the retina, thus producing a thick carpet of GFAP+ cells close to the exit of the optic nerve [4]. Furthermore, over-expression of PDGF-B under control of the rhodopsin promoter also enhances astrocyte proliferation in the retina [18]. In this case, folding of the retina occurs, a phenomenon also observed in MBP-PDGF-B transgenic mice that, in addition, exhibit disorganization of capillaries in the retina [19].

HV cells express PDGFRβ [16] but not PDGFα [15]. In the case of Arf-deficient mice with a persistent HV, it has been proposed that inadequate repression of the PDGFRβ promoter in mural cells stimulates their proliferation at the expense of differentiation [20]. This proposal implies that interactions between PDGF and its receptors are important not only for the maintenance, but also for regression of the HV.

To characterize the effects of over-expression of PDGF in neural progenitor cells on the developing retina, we developed transgenic mice that express PDGF-B under the control of a nestin enhancer element, that is active in progenitor cells during development, but not in adult neural stem or progenitor cells [21]. These nes/tk-pdgfb-lacZ mice are viable and fertile but develop enlarged lateral ventricles and mild behavioral abnormalities [22]. In the present investigation we report that the eyes of these mice display severe defects in retinal development.

## Materials and Methods

### Animals and genotyping

Our experimental model, nes/tk-PdgfB-lacZ mice over-express a 730-bp PDGF-B cDNA of human origin under control of the second intron enhancer of the human nestin gene (nes) joined to a basic thymidine kinase promoter (tk). An internal site for ribosomal entry (IRES) and a LacZ reporter gene were cloned downstream of the PGDF-B gene. The nes element enhances expression in nestin-expressing cells during embryonic development, but not in the adult cells that express this protein [21]. The generation and characterization of the nes/tk-PdgfB-lacZ strain have been described elsewhere [22] and all animals used here belongs to the #310 line described in this earlier report.

A back-cross to C57Bl/6 mice for 5 generations was performed prior to the onset of the experiment. All animal protocols were pre-approved by the local ethics committee for laboratory animals. Embryonic day 0.5 (E0.5) was defined on the basis of the presence of a copulatory plug in the morning and postnatal day 0 (P0) was defined as the morning of the day of birth. Animals were sacrificed by CO_2_ gas, cervical dislocation (pregnant dams and animals >15 days of age) or by decapitation (embryos and pups <14 days old). The transgenic mice were identified by PCR analysis as described elsewhere [19]. In brief, genomic DNA extracted from tail biopsies was subjected to PCR (95°C for 10 min, 40 cycles×[94°C for 30 sec, 55°C for 30 sec, 72°C for 1 min], 72°C for 7 min) utilizing: 5′-TGCTGCTACCTGCGTCTG as the forward primer and 5′-TTCTTCCGCACAATCTCG as the reverse primer.

### Tissue preparation and immunohistochemical analysis

For our purposes, we chose to routinely analyze the eyes and retinas of mice at the following stages of development in utero and post-natal life: E13.5, E15.5, E17.5, P1, P5, P10, P15, P20, P30, P60 and P90. On occasion, other time-points were examined as well.

To obtain cryosections, eyeballs were dissected out, fixed in 4% PFA (4°C for 15–20 minutes), washed with phosphate-buffered saline (PBS) (10 minutes at 4°C), cryoprotected in 30% sucrose (at 4°C for 3–4 hours), embedded in OCT (Sakura, Alphen aan den Rijn, The Netherlands) and frozen. 10 µm sections were cut, collected onto SuperFrost Plus objective glasses (Menzel-Gläser, Braunschwieg, Germany) and subsequently stored at −20°C or −80°C until being analyzed. Specimens were either stained with hematoxylin and eosin (H&E) or processed for immunostaining. For immunohistochemical staining, these sections were first re-hydrated in PBS for 15 minutes and then blocked and permeabilized for 30 minutes in blocking solution (PBS, 1% FCS and 0.1% Triton-X supplemented with 0.02% thimerosal for purposes of preservation). Thereafter, the sections were incubated with primary antibodies in blocking solution overnight at 4°C and subsequently with secondary antibodies in the same solution for 2–4 hours at room temperature. Following each such incubation with antibodies, the samples were rinsed 3 times with PBS for 5 minutes each. Finally, the sections were then mounted and coverslipped using VectaShield HardSet (with or without DAPI, H-1400/H-1500, Immunkemi, Järfälla, Sweden).

In the case of retinal flatmounts, eyeballs were fixed in 4% PFA (at 4°C for 3 hours) prior to dissecting out the retinas, which were then washed for 10 minutes in PBS and subsequently stored in blocking solution at 4°C until being analyzed. For staining, the retinas were incubated successively with primary and secondary antibodies overnight at 4°C, with washing in PBS (4–6 times, 1 hr each time after each such incubation). After the final wash, perpendicular cuts were made at the peripheral margins of the retina to facilitate flattening. Finally, the retinas were mounted onto SuperFrost Plus objective glasses and coverslipped as described above.

### Antibodies and image acquisition

A list of the primary antibodies employed is presented in Table S1.

The secondary antibodies (diluted 1∶200) were obtained from Vector Laboratories, Jackson Immunoresearch Laboratories and Molecular Probes. Samples were examined under a Zeiss Axioplan2 microscope and images acquired using the Axiovision software. These images have been formatted, resized, combined, enhanced and arranged for publication in Axiovision or Adobe Photoshop.

### Cell counting in P10 retinas

For quantification of the proportions of different cell populations in the INL and GCL, P10 retinas were co-stained for Pax6 and Isl1 and the resulting fractions of Isl1+ only, Pax6+ only and Isl1+/Pax6 double positive cells were manually counted in images taken using the 20× or 10× objective. Four images from two individuals were counted and the fractions of cells single or double positive for the markers were expressed as percentages of the total number of stained cells per retina image. Student's t-test was performed to test for significant differences between wild-type and transgenic mice, where p<0.05 was considered significant.

### Staining for X-Gal

In order to monitor the localization and temporal pattern of the activity of the transgene via the LacZ reporter, cryosections from E13.5- P90 animals (prepared as described above) were stained for X-gal. After re-hydration in PBS for 10 minutes and post-fixation in PBS containing 1% formaldehyde, 0.2% glutaraldehyde, 0.02% NP40, 2 mM MgCl_2_, and 5 mM EGTA, pH 8.0, for 15 minutes at room temperature, these sections were washed 3×5 minutes in X-Gal wash buffer (PBS containing 2 mM MgCl_2_, 0.01% Na-deoxycholate and 0.02% NP40). Visualization of X-galactosidase activity was carried out by incubation at 37°C for 4 hours in X-Gal wash buffer supplemented with 1 mg X-Gal/ml, 5 mM K_3_Fe(CN)_6_ and 5 mM K_4_Fe(CN)_6_. The sections were washed 2×5 minutes in X-Gal wash buffer, then washed once in PBS and finally mounted in Entellan (VWR, Gothenburg, Sweden).

### Determination of proliferation utilizing EdU

To examine proliferation during the earliest phases of transgene activation, pregnant dams carrying E18.5 pups were injected intraperitoneally with 200 µg of the nucleotide analogue 5-ethynyl-2′-deoxyuridine (EdU) and the pups analyzed 2 days later, at P1. The eyes were fixed, frozen and sectioned as described above and the Click iT EdU imaging kit (C10083/C10337, Invitrogen, Paisley, UK) employed in accordance with the manufacturer's protocol to visualize cells that had incorporated EdU during S-phase [23].

### Measurement of intraocular pressure

The intraocular pressure (IOP) in 3–4 months-old mice was measured using the TonoLab rodent tonometer (Icare Finland Oy, Espoo, Finland) in accordance with the manufacturer's instructions, following sedation by i.p. injection of Rompun (10 mg/kg, Bayer Animal Health, Copenhagen, Denmark) and Ketalar (60 mg/kg, Pfizer, Sollentuna, Sweden). For each mouse the IOP in both eyes (with the exception three transgenic mice in which one eye was too small to allow reliable measurements) was determined consecutively 6 times and the means for the transgenic (9 animals, 15 eyes measured) and wild-type mice (11 animals, 22 eyes measured) analyzed for statistically significant differences utilizing the Mann-Whitney test.

### Administration of STI571

STI571 was administered (100 µg/g body weight, diluted in dH_2_O) by gavage once daily during three different time intervals: the first group received this inhibitor between E17.5 and P0 or P1 (samples taken for analysis at P1 or P2, respectively), the second group from P0–P4 (samples being taken for analysis on P5), and the third group between P7 and P14 (samples taken on P15). The body weights and general health of the mice was monitored daily. The eyes were fixed, frozen and sectioned as described above.

### Statistical analyses

The data were analyzed in Excel and graphs created in GraphPad Prism (v3.02, GraphPad Software Inc., La Jolla, USA).

## Results

### Morphological alterations in eyes of mice over-expressing PDGF-B

**Figure 1:**
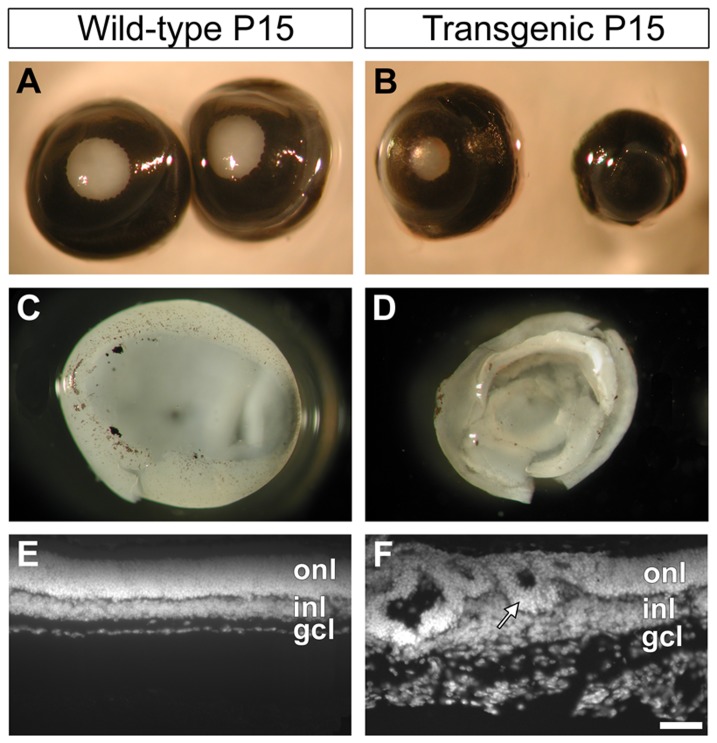
Morphological alterations in eyes of mice over-expressing PDGF-B. Eyes from wild type (A) and transgenic (B) mice on postnatal day 15 (P15) were photographed after dissection. Folding was observed in the transgenic (D) retina, compare to wild type (C). The eye-pairs showed in (A) and (B) are from the same individual, respectively, and the retinas shown in (C) and (D) were dissected from the eyes shown in (A) and (B, large eye). Histological examination (DAPI nuclear stain) revealed a disorganization of retinal lamination (F) not observed in wild type eyes (E). Outer nuclear layer (onl), inner nuclear layer (inl), ganglion cell layer (gcl), Scale bar 50 µm.

In the transgenic mice employed, PDGF-B is over-expressed in nestin-expressing cells of the CNS during development [22]. The macroscopic appearance of the nes/tk-PdgfB-lacZ mice and their wild type littermates was generally similar, except that the transgenic mice displayed enlarged lateral ventricles, smaller eyes and had abnormal behavior as described previously [22]. At the time of eye opening, the transgenic mice displayed uni- or bilateral reduction in the size of their eyes and/or irises (Fig. 1A–D) and we frequently observed blood inside the eyeball in transgenic mice (Fig. S1A). Retinal folding with frequent attachment to the lens was observed in all the transgenic animals after birth (Fig. 1D). With increasing age of the transgenic mice, the retinas degenerated progressively and after 2–3 months they were markedly thinner than those of the wild-type animals (Fig. S1B–D). Around one year of age, the retinas had degenerated completely and consisted of only a thin sheet of cells (Fig. S1E).

Histological examination revealed a severe distortion of the transgenic retinas, with disorganization of retinal lamination discernable already in the earliest days of post-natal life. One prominent feature of these retinas was rosette-like structures in the outer nuclear layer (ONL) containing the nuclei of photoreceptors (Fig. 1F, arrow), which appeared between P5 and P10 (Fig. S2) The other nuclear layers of transgenic retina; the inner nuclear layer (INL) and the ganglion cell layer (GCL) were also distorted, with local variations in thickness and folding. In general, the nuclear and plexiform layers could be distinguished from one another although with a varying degree of distortion (Fig. 1F). Among the general laminar disarray, local regions in some retinas appeared normal. The progressing deterioration of the neural retina was visualized by H&E staining (Fig. S2).

### The temporal pattern of transgene expression in the developing retina

**Figure 2:**
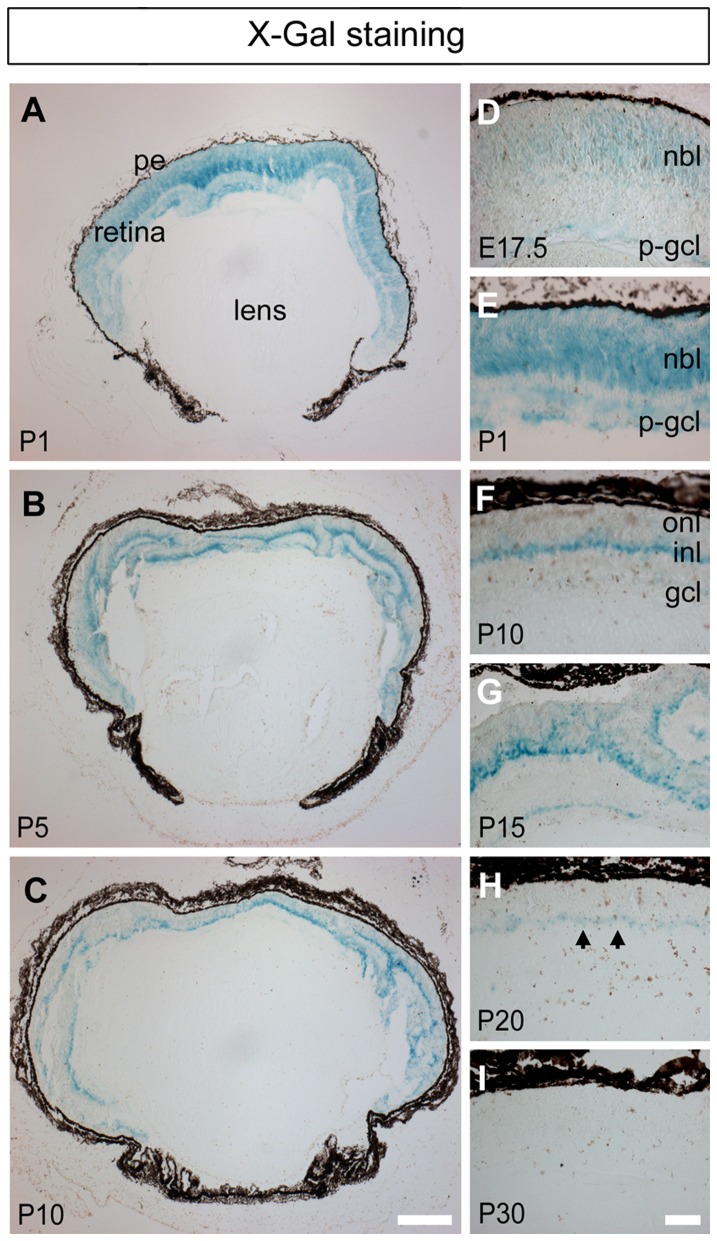
Expression of the transgene in the developing retina. X-gal staining in sectioned eyes from transgenic mice at different postnatal days (P1–P30). Pigmented epithelium (pe), neuroblastic layer (nbl), putative ganglion cell layer (p-gcl), inner nuclear layer (inl). Arrowheads in H depict X-gal staining in the inl at P20. Scale bar A–C 200 µm, D–I 50 µm.

We have previously shown that X-gal staining in this mouse strain faithfully depicts transgenic PDGF-B expression [22]. In order to monitor changes in the expression of the transgene with time, the LacZ reporter gene and staining of X-gal in sectioned tissue were therefore employed (Fig. 2A–I). In contrast to the brain and spinal cord, where X-gal staining is most intense between E10.5 and E14.5 [22] retinal expression occurred later during development. In this tissue, staining first appeared as a faint signal distributed throughout the neuroblastic layer (NBL) and in some cells within the putative ganglion cell layer (GCL) on E17.5 (Fig. 2D). On P1 the pattern of staining was similar, but much darker (Fig. 2A and 2E) and between P5 and P15, the robust staining persisted, albeit now restricted to a band of cells located in the middle of the INL (Fig. 2B, 2C, 2F and 2G; note the localization of X-Gal staining in the ‘near-normal’ region shown in Fig. 1F). By P20, the intensity of the staining, although still detectable, had attenuated significantly (Fig. 2H, arrowheads) and after P30 no X-Gal staining could be seen (Fig. 2I and not shown). We conclude that transgene expression in the retina commenced on E17.5 and attained its maximal level between P1 and P15, before declining on P20.

### Neural development and cell death in the retinas of mice over-expressing PDGF-B

**Figure 3:**
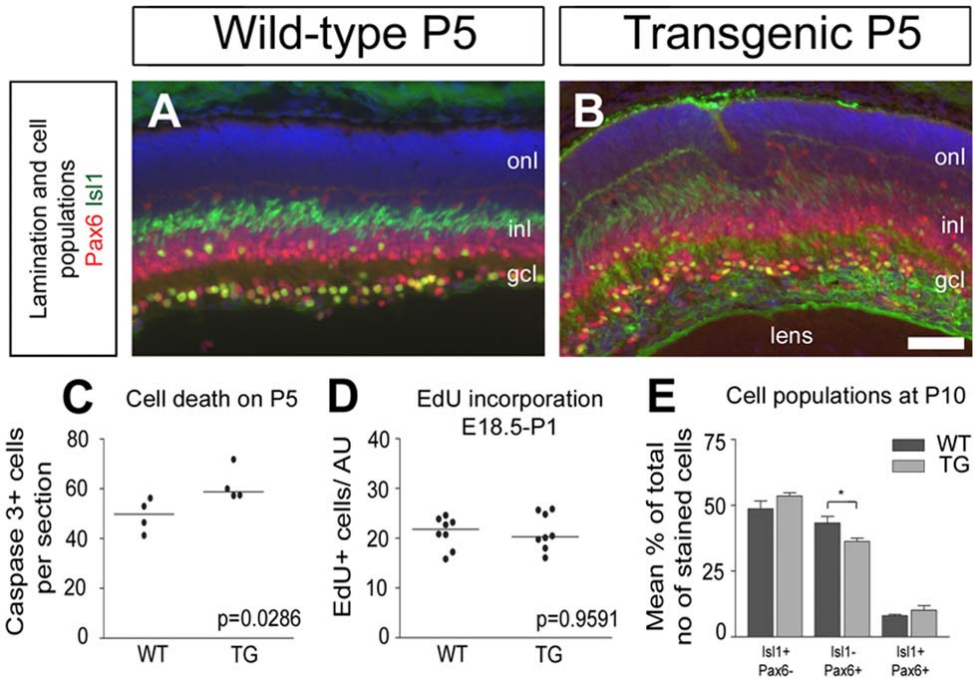
Cell lineage development, cell death and proliferation in the transgenic retina. Evaluation of cell populations in wild type (A) and transgenic (B) retina sections on postnatal day 5 (P5). Quantification of programmed cell death by caspase 3-positive cells on P5 (C). Quantification of cell proliferation by EdU incorporation between E18.5 and P1 (D). Quantification of Isl1+ or Pax6+ single positive cells or Isl1/Pax6 double positive cells in P10 retina (E) reveal a significant decrease (*, p = 0.01) in the number of Isl1-/Pax6+ cell population in transgenic retina. Outer nuclear layer (onl), inner nuclear layer (inl), ganglion cell layer (gcl), Scale bar 50 µm.

Since the onset of expression of the transgene in the nes/tk-PdgfB-lacZ retina is concurrent with retinal neurogenesis, lamination and developmental cell death we examined whether over-expression of PDGF-B affected these processes. First, we analyzed the generation of the different types of retinal neurons and their laminar positions by staining with antibodies for the five major markers: Pax6 (expressed by horizontal cells, HCs, amacrine cells, ACs, and retinal ganglion cells, RGCs), Isl1 (bipolar cells, BPs, RGCs), calbindin (HCs and certain ACs), neurofilament 165 kD (neurites in the plexiform layers) and the photoreceptor (PR) markers recoverin and rhodopsin. Co-labeling with Pax6 and Isl1 revealed that over all, the different cell types were present in the retina, and despite the apparent laminar distortion at the correct positions relative to one another (Fig. 3A–B).

To determine if there were detectable differences in cell number, we next analyzed markers of proliferation and apoptosis. We first used an antibody directed against cleaved caspase 3, and found that in P5 eyes the number of labeled cells in the transgenic retinas was increased by 10% compared to corresponding normal tissue (Fig. 3C). We also analyzed the effects of over-expression of PDGF-B on the proliferation by using the proliferation marker Ki67 (not shown) and incorporation of the nucleotide analogue EdU (Fig. 3D). Pregnant mice were injected with EdU on E18.5 and analysis was performed two days later. No differences between transgenic and wild-type mice were discernable at the ages tested (Fig. 3D). Finally, by counting cells expressing Isl1 and/or Pax6 in P10 retinas we estimated that here was a slight but significant decrease in the Isl1-/Pax6+ cell population (Fig. 3E).

### Altered photoreceptor development and maturation by over-expression of PDGF-B

**Figure 4:**
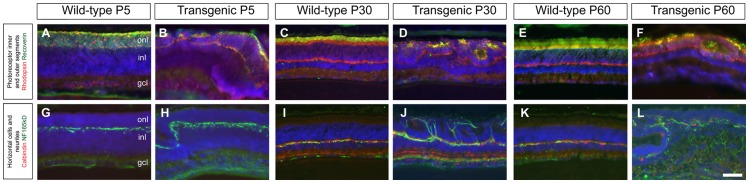
Analysis of makers for photoreceptors and horizontal cells. Wild-type (A, C, E, G, I, K) and transgenic (B, D, F, H, J, L) retinas were cross-sectioned and stained with the antibodies indicated at ages P5, P30 and P60. Scale bar A–L 50 µm. Outer nuclear layer (onl), inner nuclear layer (inl), ganglion cell layer (gcl).

Analysis of rhodopsin and recoverin immunoreactivity at P5 revealed that transgenic retinas stained differently from the wild-type. Rhodopsin staining appeared displaced relative to its normal position (Fig. 4A–B). In the adult, the staining of the inner and outer segments of the photoreceptors in nes/tk-PdgfB-lacZ mice was irregular and frequently restricted to isolated patches on the outer side of the outer nuclear layer (ONL) or located within the lumen of the rosettes (Fig. 4C–F)

Horizontal cells (HC) make synapses with photoreceptors within the outer plexiform layer (OPL) of the retina. Staining for the HC markers NF165 kD and calbindin revealed that the appearance of cells and neurites in transgenic retinas were normal up until P5 (not shown), but exhibited abnormalities thereafter (Fig. 4G–L). On P10, a time when many synapses are formed in the mouse OPL [24], the neurites of transgenic HC developed ectopic extensions outside of the OPL (not shown). These extensions reached into and across the ONL and on P30 they surrounded photoreceptor rosettes (Fig. 4I–J). These ectopic HC neurites persisted, showing little or no sign of regression even on P60 (Fig. 4K–L).

### Glial activation in the retinas of mice over-expressing PDGF-B

**Figure 5:**
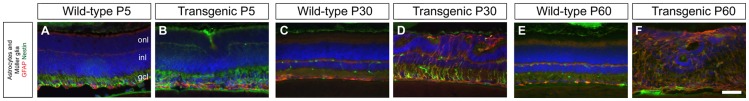
Analysis of makers for glial cells. Wild-type (A, C, E) and transgenic (B, D, F) retinas were cross-sectioned and stained with GFAP (red) and Nestin (green) at ages P5, P30 and P60. Scale bar A–L 50 µm. Outer nuclear layer (onl), inner nuclear layer (inl), ganglion cell layer (gcl).

**Figure 6:**
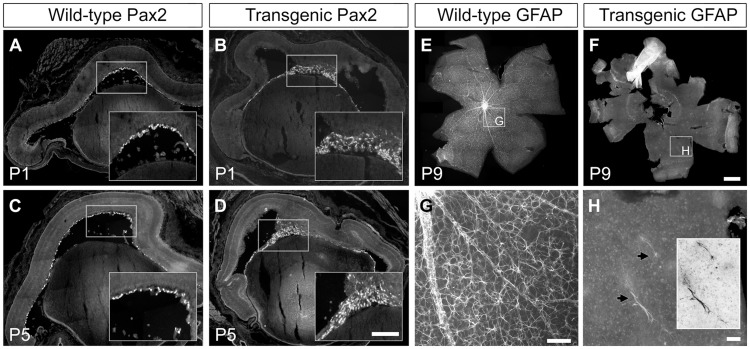
Glial activation in the PDGF-B transgenic retina. Retinas from wild-type (A, C) and transgenic (B, D) mice were stained for Pax2 on postnatal day 1 (P1) and P5 to depict astrocyte precursors. Mature astrocytes were visualized with GFAP antibodies on retinal flat-mounts from wild-type (E) and transgenic mice (F). Panels G and H are magnifications of E and F. The insert in H shows astrocyte morphology in the transgenic retina (the same cells are indicated by an arrow). Scale bar A–D 200 µm, insets 100 µm, E–F 1 mm, G–H 100 µm.

Müller glia and astrocytes are the major two types of glia cells in the murine retina. Glial fibrillary acidic protein (GFAP) is widely used as a marker for the latter, whereas nestin is commonly employed to identify activated Müller glia cells. Ischemia or other retinal damage triggers the expression of both nestin and GFAP in Müller cells [25]. Since antibodies towards Pax2 stain the precursor cells to retinal astrocytes, a combination of antibodies directed against nestin, GFAP and Pax2 were used to monitor glial development in the transgenic retina. Although the expression of nestin by Müller glia cells in transgenic retinas appeared relatively normal during early postnatal development (not shown), on P5 GFAP-positive cells were more abundant in transgenic retinas than in wild-type tissues (Fig. 5A–B). In the transgenic retinas Pax2+ and GFAP+ cells did not spread out evenly on the nerve fiber layer between E17 and P10 as wild type astrocytes do [26]. Instead, on P1 and P5, astrocyte precursors staining positively for Pax2 remained at the vitreal side, and did not populate the retina (Fig. 6A–D). Although the transgenic retina contained scarce GFAP-expressing cells (Fig. 6F, 6H) with normal morphology (insert in Fig. 6H), these cells were not evenly distributed in contrast to the wild type retina (Fig. 6E, 6G). Signs of glial activation in transgenic retinas became more manifest with time: elevated expression of GFAP in the inner parts of the retina and of nestin on P30 (Fig. 5C–D) indicates the presence of either ischemia and/or mechanical forces exerting traction on the retina. At the age of P60, the glial activation was exacerbated (Fig. 5E–F).

### Over-expression of PDGF resulted in failure to vascularize the retina

**Figure 7:**
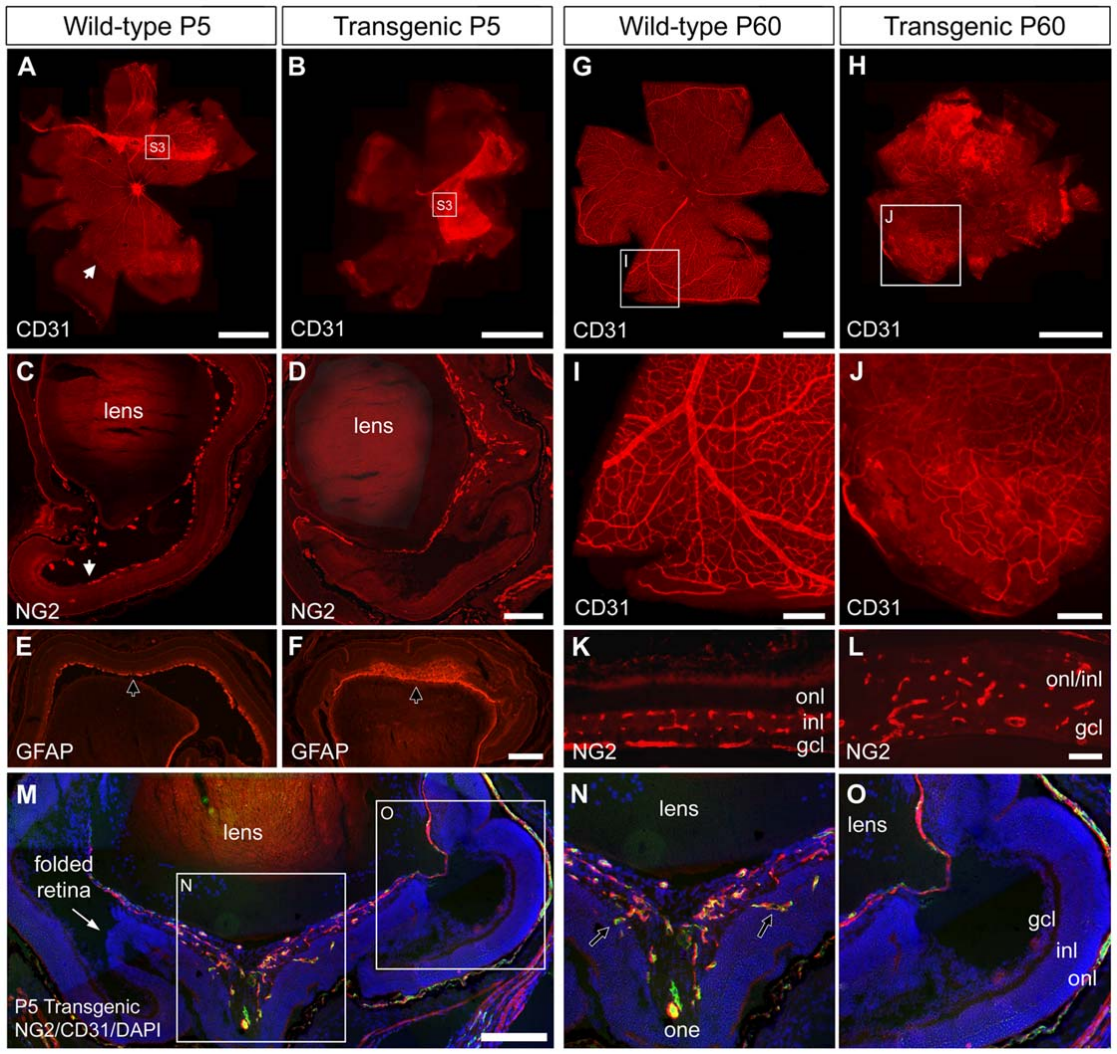
Failure to vascularize the retina. Staining of cross-sections and flat-mounts with antibodies against CD31, NG2 and GFAP on postnatal day 5 (P5, (A–F, M–O) and P60 (G–L). The white arrowhead in A and C indicates the border of CD31-positive cells in a control mouse. Note their absence in the transgenic retina (B and D). NG2, CD31 and GFAP staining is localized to the retrolental cell mass, indicative of the hyaloid, in transgenic retinas (D, F, M–O) compared to wild-type retinas (C, E). Irregular CD31+ cells had developed at P60 in the transgenic retina (H, J, L). Wild-type control (G, I, K). M–O: The same P5 transgenic retina as shown in D, stained for NG2 (red) and CD31 (green) and DAPI (blue). Tractional forces on the retina have caused it to fold (thin white arrow in M) by sprouting ectopic and irregular blood vessels (black arrows in N) that infiltrates the retina at various depths. Outer nuclear layer (onl), inner nuclear layer (inl), ganglion cell layer (gcl), optic nerve exit (one). Scale bar A and B 1 µm, C–F 200 µm, G–H 1 mm, I–J 200 µm, K–L 50 µm and M 200 µm.

During postnatal development in the mouse, retinal vascularization takes place concomitantly with regression of the hyaloid vasculature, which initially supplies the eye with oxygen. Upon staining cross-sections and flatmounts with antibodies against CD31 (an endothelial cell marker) and NG2 (a pericyte marker) (Fig. 7) on P5 virtually no CD31+ or NG2+ cells were detected in the retinas of transgenic mice, indicating complete failure of retinal vascularization and angiogenesis (Fig. 7A–D, 7M–O). In their wild-type littermates (Fig. 7A) newly formed blood vessels had covered more than 70% of the retina by this time (arrowhead point at the border of CD31+ cell migration). In contrast, remnants of the embryonic vasculature was more abundant in the transgenic mice (Fig. 7A–D and Fig. S3). In addition, GFAP staining showed that the transgenic astrocyte precursors did not spread out to populate the retina in a normal fashion (Fig. 7E–F). Consequently, there was a delay in population of the retina by vascular cells, and the network of vessels in the inner retina of transgenic mice that finally formed was abnormal (Fig. S4).

### Abnormal development of the retinal capillary network

At P20, the transgenic retina was vascularized, although this took place in an irregular fashion (Fig. S5). At P60, a notable feature of the transgenic vascular network was the absence of large trunk vessels (compare Fig. 7H, 7J with 7G, 7I), a finding confirmed by the low level or absence of α-SMA immunoreactivity, characteristic of smooth muscle cells on arteries (Fig. S6). The NG2 and CD31 staining patterns revealed that at P60, vessels resembling capillaries of varying diameters and thickness grew irregularly into the retina (Fig. 7H, 7J), populating the different retinal layers in an apparently random manner (Fig. 7L). Staining of retinal whole mounts for CD31 and NG2 at P9 to visualize interactions between endothelial cells and mural cells show that there was some pericyte coverage of transgenic vessels albeit irregular (Fig. S7). Some NG2+ cells were attached to CD31+ cells while others were found separated from the endothelium. This demonstrated that over-expression of PDGF did not completely abolish the interactions between endothelial and mural cells.

### The transgenic mice exhibited reduced intraocular pressure

**Figure 8:**
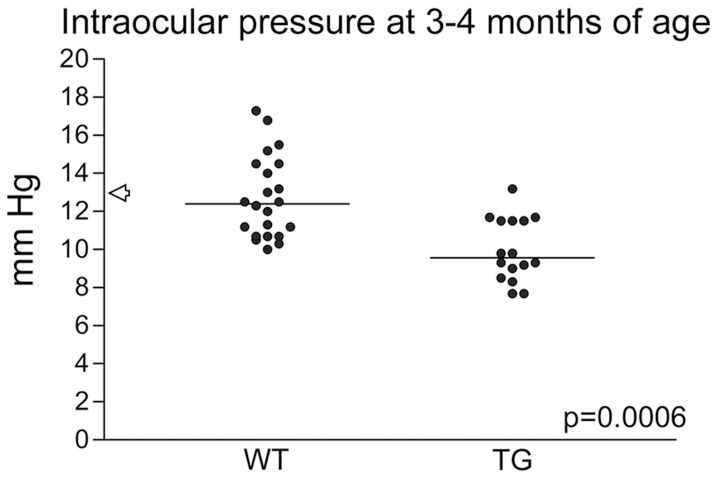
Transgenic mice exhibit reduced intraocular pressure. Measurement with a rodent tonometer revealed significantly lower pressure in the eyes of transgenic (TG) than in wild-type (WT) animals. The arrow indicates the reference value for C57Bl/6 mice.

We observed that blood and other fluid often (∼80% of the mice examined) oozed out of nes/tk-PdgfB-lacZ eyes following puncture for retinal dissections, indicating that the retinal blood vessels were leaky and perhaps that the circulation of ocular fluids in these mice is abnormal (Fig. S1A). The apparent lack of arteries also suggested that the ability to stabilize circulatory pressure in the eyes should be reduced. When the intraocular pressure in 3–4 month-old transgenic and wild-type mice was determined using a rodent tonometer, the transgenic animals displayed significantly lower pressure (9.55 versus 12.4 mmHg for the wild-type, p = 0.0006, Mann Whitney test) (Fig. 8).

### Partial restoration of astrocyte precursor migration by the tyrosine kinase inhibitor STI571

In an attempt to pinpoint the period during which over-expression of PDGF affected the formation of various cells in the retina and possibly even reverse the defects observed, the mice were administered the tyrosine kinase inhibitor STI571 (Imatinib, Glivec or Gleevec) systemically by oral gavage once daily during three different intervals: from E17.5 until P0 or P1 with samples taken for analysis on P1 or P2, respectively, from P0–P4 (samples taken for analysis on P5), or between P7 and 14 (samples taken on P15). STI571 blocks signaling via PDGF receptors, and in addition, inhibits other tyrosine kinases, such as c-Kit and Bcr-Abl [27]. We found that E17.5 was the earliest time point at which pregnant females could be treated with STI571 since earlier treatment resulted in the premature birth of non-viable pups.

**Figure 9:**
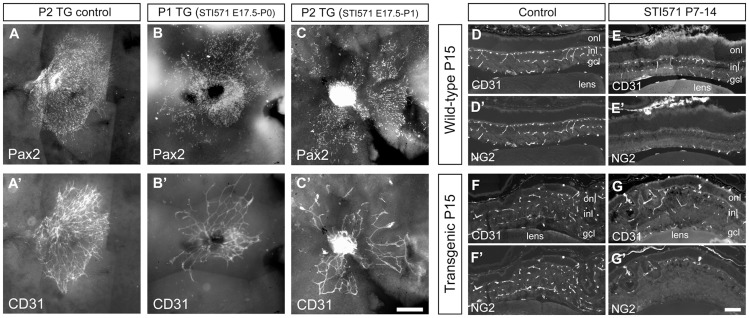
Partial restoration of Pax2+ and CD31+ migration after STI571 treatment. Retina flat-mounts of postnatal day 1 (P1) or P2 mice whose mother received the tyrosine kinase inhibitor STI571 (Glivec) from E17.5 to birth (B, B′), or from E17.5 to birth and at P1 (C, C′) and P2 control mice (A, A′). In panels D–G, the inhibitor was administrated to wild-type (D, D′, E, E′) or transgenic (F, F′, G, G′) mice between P7 to P14. Cross sections of retina were stained with antibodies to CD31 and NG2. Outer nuclear layer (onl), inner nuclear layer (inl), ganglion cell layer (gcl). Scale bar A–C, A′–C′ 200 µm, D–G, D′–G′ 100 µm.

STI571 treatment during the late embryonic period partly restored colonization of the transgenic retina by astrocyte precursors (Fig. 9). In untreated transgenic P2 mice the Pax-2 positive and CD31+ cells exhibited the characteristic compact and irregular cell mass that associates with the HYV (Fig. 9A and 9A′, see also Fig. 7F). However, in STI571 treated pups Pax2-positive astrocyte precursor cells had not been trapped by the hyaloid to the same extent, and were more spread apart (Fig. 9, compare 9A with 9B and 9C). Concomitantly, CD31+ cells also had an organization that was less dense than in non-treated mice (Fig. 9, compare 9A′ with 9B′ and 9C′). Suppression of PDGF signaling in wild-type by STI571 during the early postnatal period (P0–P4) inhibited colonization of the retina by vessels (Fig. S8A–B). However, no difference between treated and un-treated transgenic mice with respect to CD31 staining was detected (Fig. S8C–D).

P7–P14 was the latest time window during which phenotype rescue was attempted. The hyaloid in un-treated nes/tk-PdgfB-lacZ mice was very often attached to both the lens and retina, making it difficult to dissect out these structures damaging the retina. Moreover, prominent folding of the retina (Fig. 1D) often made flatmounting challenging. In contrast, on P15, the lens of transgenic mice treated with STI571 was easier to dissect out, and indeed, actually fell out not being stuck in the hyaloid, indicating partial reversal of these effects. In the case of both transgenic and wild-type mice, treatment with this inhibitor resulted in less coverage of deep CD31+ vessels by NG2 positive cells (Fig. 9D–G, 9D′–G′).

## Discussion

We report here that in mice expressing elevated levels of PDGF-B in nestin positive cells of the retina during development, astrocytes fail to populate the retina and vascular progenitors do not colonize this tissue to produce a network of vessels. Interestingly, with the exception of their eyes, nes/tk-PdgfB-lacZ mice do not exhibit any profound alteration in phenotype [22]. The transgenic eyes were smaller than normal with an iris of a reduced diameter and with frequent occlusions. The transgenic retinas had folds and adhered to the back of the lens, forming a retrolental mass that made dissection difficult. This morphology was accompanied by frequent bleeding and by the age of one year the transgenic retina had deteriorated completely. Many of these defects in the eye are likely to be associated with abnormal traction forces within the structures that eventually also leads to retinal detachment (Fig. 7M–O). Patients with persistent fetal vasculature syndrome (PFVS) [28] display similar problems [1] with a large individual variability that was also seen among the nes/tk-PdgfB-lacZ mice. Many of the transgenic mice developed uni-lateral microphtalmia (around 40–50% pups per litter) and/or a cataract. These abnormalities in retinal structure of the transgenic mice are similar to PFVS, which results from failure to degrade the hyaloid vasculature.

The crucial role of PDGF signaling in connection with the normal formation of retinal blood vessels is well-established [29]. The functions of PDGFRα-signaling include regulation of the migration and proliferation of astrocyte precursor cells, while signaling by PDGFR-β recruits mural cells, which allows for their proper attachment to the endothelial cells of the blood vessels [30] [14]. Seo and coworkers [31] have proposed that gain-of-function mutations in PDGF-B exert more profound effects on retinal development than mutations in PDGF-A, due to the ability of the former to activate both α and β receptors, present on different types of cells. This suggestion was confirmed in studies involving overexpression of different isoforms of PDGF in photoreceptors [32] and further supported by the findings of Vinores et al [18]. In our previous study [19] involving transgenic expression of PDGF-B under control of the myelin basic protein promoter a disorganized neural retina with an under-developed capillary network was observed. The transgenic mice employed here display more severe alterations in eye phenotype, probably because the transgene is expressed more widely, both spatially and during the period of development examined. By binding to both the α and β receptors [29] the B isoform of PDGF could affect the hyaloid as well as astrocytes [16] and pericytes [30], cells that form and provide support for the vasculature during a critical period of retinal development.

The defects in lamination discernible shortly after birth are consistent with the onset of transgene expression on E17.5 with peak expression on P1. Since no major effects on the representation of retinal cells, on their relative spatial orientation or on their proliferation were seen, despite the overall disorderly structure, we conclude that the transgene expression did not affect the general formation, differentiation or migration of retinal progenitors.

The elevation of the number of caspase positive cells observed on P5 occurred at a time when this tissue normally undergoes developmental cell death [33]. Cell death is part of the normal development of a functional nervous system and half of all postmitotic retinal ganglion cells die during the 2 first post-natal weeks in mice [34]. This period of cell death is associated with trophic interactions within the retina and with the central targets for the retinal ganglion cells, and the extent of death is determined in part by what connections the cells can establish. The fact that we see more apoptosis in the transgenic mice during this period suggests that the increased cell death is associated to the structural changes in the retina and is not a direct effect of the transgene. Moreover, the deterioration of the retina, as seen after one year, is probably not related to this increased apoptosis but rather related to the malfunctioned blood supply to the eye.

The inner and outer segments of the photoreceptor layer in the transgenic animals did not develop normally. The rhodopsin localized in patches or inside the rosette formations and the horizontal cells, which normally form synapses with photoreceptors [35], developed ectopic neurites that extended outside the outer plexiform layer and into rosettes. This pattern persisted in the adult retina for several months indicating that these cells may have formed synapses with the PR, despite their anomalous location.

The up-regulation of nestin and GFAP in Müller glia cells indicated that the transgenic retina experienced a gliotic response that is likely to be triggered by ischemia due to the missing retinal vasculature. Mechanical traction may be an alternative explanation since Müller glia is known to exert tractional forces when stimulated by PDGF in vitro [36]. It seems likely that traction also occurred in the transgenic retinas. During normal retinal vascularization, astrocyte precursor cells migrate from the optic nerve to populate the inner portion of the retina, where they form a scaffold on which vascular progenitor cells can migrate and form vessels [37]. In the nes/tk-PdgfB-lacZ mice most of the Pax2/GFAP positive cells remained associated with the vitreal side of the retina, and consequently, no astrocyte network on which vessels could expand was laid down. The lack of proper formation of new vessels was also apparent from the staining of CD31 and NG2 in both flat-mounts and cross-sections of the nes/tk-PdgfB-lacZ retina. This staining demonstrated defective vascularization, with few capillary-like structures, in a seemingly random fashion and devoid of large trunk vessels. Our results suggest that over-expression of PDGF-B delayed regression of the hyaloid, and prevented APCs from migrating and spreading across the retina, which in turn prevented endothelial and mural cells from populating the retina to form a normal vascular network. Endothelial cells recruit pericytes by secreting PDGF-B [38] and it is well established that pericytes require PDGF-B to remain attached to the vessel wall [30]. The opposite situation, i.e. an excess of PDGF-B is apparently not as detrimental, since many transgenic pericytes remained attached to the endothelial cells. Therefore, the abnormal vessel properties cannot mainly be related to altered coverage by mural cells.

Intraocular pressure (IOP) in mice varies between strains [39], underscoring the importance of using non-transgenic littermates as controls when congenic strains are not available. The mice examined here had been back-crossed with C57Bl/6 mice for 5 generations, but the remaining contribution of the CBA genome is unknown. Nonetheless, the wild-type control mice, which were always siblings, exhibited an IOP close to the reference value for C57Bl/6 [40]. The attenuated IOP in our transgenic mice is consistent with our hypothesis that the perfusion of the eye is severely affected in the transgenic animals. The lack of proper retinal vessels prevented these mice from controlling the ocular circulation. Furthermore, traction on the ciliary body can lead to acute and chronic hypotony, followed by retinal detachment, as has been reported in patients with PFVS [28].

The effect of STI571 on the retinal phenotype of the transgenic mice depended on the time when this inhibitor was administrated. Administration prior to E17.5 caused abortion, demonstrating that PDGF signaling, and/or other tyrosine kinases sensitive to STI571 are critical for embryo survival. From E17.5 to the time of birth, exposure to STI571 did not affect the birth of live offspring. Since this time period coincided with the onset and early phase of transgene expression in the eye, the earliest effects of transgenic PDGF could be monitored. The timing of inhibition was of importance for APC maturation and spread [41]. APCs were less compacted upon treatment with STI751, which apparently allowed CD31-positive cells to follow, since these cells were also less tightly packed in the presence of the inhibitor. However, the retinal vascularization was not greatly improved despite the better APC colonization. The observation that vessel distribution was not influenced at all by administration of STI571 from birth to P4 suggests that at this point it is too late to reverse the abnormal development of the vascular network. In contrast, if STI571 was administered to the mice during the second week of postnatal life, the hyaloid regressed in part. Thus, even though STI571 blocks all PDGF signaling, not only that originating from the transgene, we could discern partial rescue of the phenotype with this inhibitor.

The present investigation reveals that timely regression of the hyaloid vasculature and development of the adult retinal vascularization are prevented by over-expression of PDGF-B in nestin expressing cells during development (Fig. S9). Failure of astrocyte precursors to form a scaffold precluded retinal vessel formation and gave rise to a defective retina that deteriorated with time. We also showed these mice to be useful for testing pharmacological intervention by the ability of a small-molecular inhibitor to partially restore retinal vascularization.

## Supporting Information

Figure S1 **Intra-ocular bleeding and retinal deterioration over time.** (A) Photograph of a P15 eyeball from a transgenic mouse. Upon dissection, transgenic eyes frequently displayed intra-ocular bleeding, which shows leakiness of vessels, as exemplified by this P15 eye. Retinal cross-sections (B–D) from postnatal day (P) 90 mice stained for DAPI. C and D show eyes from different mice indicating the variability between individuals. (E) Retina dissected from a transgenic P304 mouse reveals that the retina has deteriorated to a thin irregular sheet. Outer nuclear layer (onl), inner nuclear layer (inl), ganglion cell layer (gcl).
(TIF)

Figure S2 **Hematoxylin/eosin staining of wild-type and transgenic retina.** Postnatal day 1 (P1), P5, P10, P15, P20 and P30 with magnifications of the boxed areas of transgenic retinas shown on the right. Arrow in P5 points to the ONL where no obvious signs of rosette formation are seen. Arrow in P10 points to rosette formation in the ONL. Boxed region in P15 shows to the retrolental cell mass surrounding the lens (not seen in the wild-type). Boxed regions in P20 and P30 indicate the large-scale distortion of retinal histology possibly caused by tractional forces exerted on the retina.
(TIF)

Figure S3 **Hyaloid vasculature express CD31 and GFAP.** Merged and split two channel images of the boxed regions in Figure 7A–B, shows hyaliod vasculature remnants at P5 at high magnification. Note the denser meshwork of CD31 (red) and GFAP (green) positivity in the transgenic hyaloid as compared to the wild-type.
(TIF)

Figure S4 **Time-course for blood vessel extension in transgenic retinas.** Post-natal day (P) 1, 5 and 9 retinas stained for CD31. At P1, the vasculature emerging from the optic nerve exit forms an irregular mass of vessels. At P5, the mass of irregular vessels have expanded, but without any apparent underlying vascular network. At P9, emerging sprouting vessels (eg. boxed area) starts to vascularize the retina, eventually giving rise to the irregular network of capillaries shown in Fig. 7J (P60) and Figure S5B (P20). The boxed area in P9 depicts the same area shown in Figure S7B.
(TIF)

Figure S5 **CD31 staining on P20.** CD31 staining of P20 wild-type (A, A′) and transgenic (B, B′) retinas reveal the failure of transgenic retinas to form normal trunk vessels. Those that have formed have very few branch points and thus appear to have limited connectivity with the extensive and disorganized capillary network of the transgenic retina.
(TIF)

Figure S6 **Lack of α-SMA immunoreactivity in transgenic retinas.** Staining with an antibody to α-SMA immunoreactivity on flat-mounts of wild-type and transgenic retinas.
(TIF)

Figure S7 **Association of mural cells with vessels in transgenic retinas.** Co-labeling of CD31 and NG2 in flat-mounts of wild-type (A) and transgenic (B) retinas on postnatal day 9 (P9) demonstrated that NG2-positive cells remained associated with vessels in the transgenic mice. Scale bar A–B 100 µm.
(TIF)

Figure S8 **Effect of STI571 treatment on postnatal day 0 (P0) to P4.** Suppression of PDGF signaling in wild-type mice by STI571 during the early postnatal period, inhibited vascularization (B). Compare to panel A without treatment. No difference was detected in transgenic mice (C, D) where CD31 stained cells remained as an irregular mass near the optic nerve exit. Scale bar A–D 500 µm.
(TIF)

Figure S9 **Comparative time series of central- and peripheral parts of wild-type and transgenic retinas.** Cross sections of embryonic day (E)17.5 and postnatal day (P) 1, 5, 10, 20 wild-type (WT) and transgenic (TG) retinas stained for NG2. The central two columns depict the low magnification images where magnified regions of central retina (left-most two columns) and peripheral retina (right-most two columns) are indicated by white boxes.
(TIF)

Table S1 **List of antibodies used in this study.**
(DOCX)
