# Supplementary figures and images for: Platelet-Derived Growth Factor Over-Expression in Retinal Progenitors Results in Abnormal Retinal Vessel Formation
Source: PLoS One. 2012 Aug 3;7(8):e42488. doi: 10.1371/journal.pone.0042488 (PMC3411765; doi:10.1371/journal.pone.0042488)

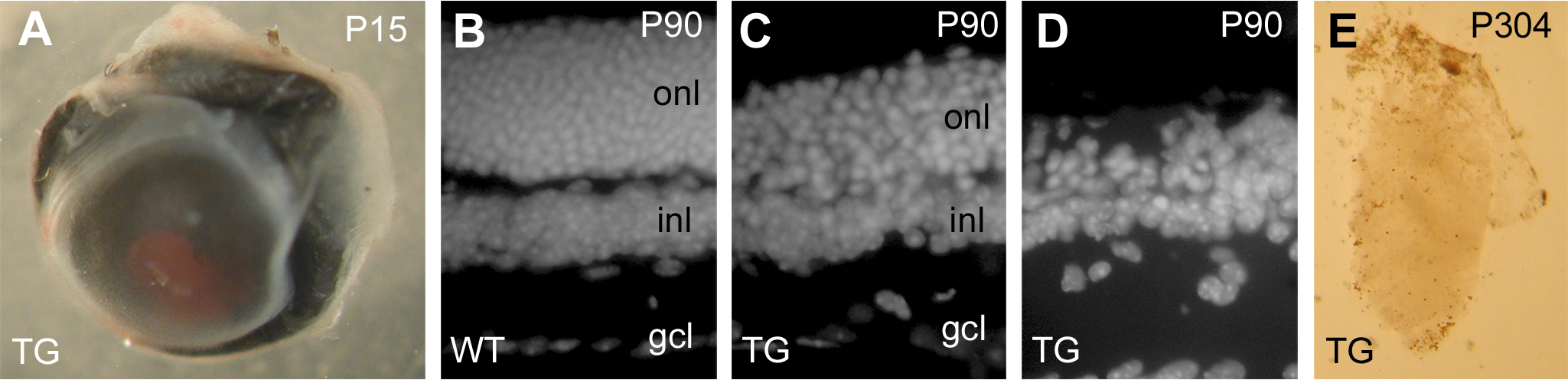

Supplement: Figure S1 — Intra-ocular bleeding and retinal deterioration over time. (A) Photograph of a P15 eyeball from a transgenic mouse. Upon dissection, transgenic eyes frequently displayed intra-ocular bleeding, which shows leakiness of vessels, as exemplified by this P15 eye. Retinal cross-sections (B–D) from postnatal day (P) 90 mice stained for DAPI. C and D show eyes from different mice indicating the variability between individuals. (E) Retina dissected from a transgenic P304 mouse reveals that the retina has deteriorated to a thin irregular sheet. Outer nuclear layer (onl), inner nuclear layer (inl), ganglion cell layer (gcl). (TIF) [file pone.0042488.s001.tif]

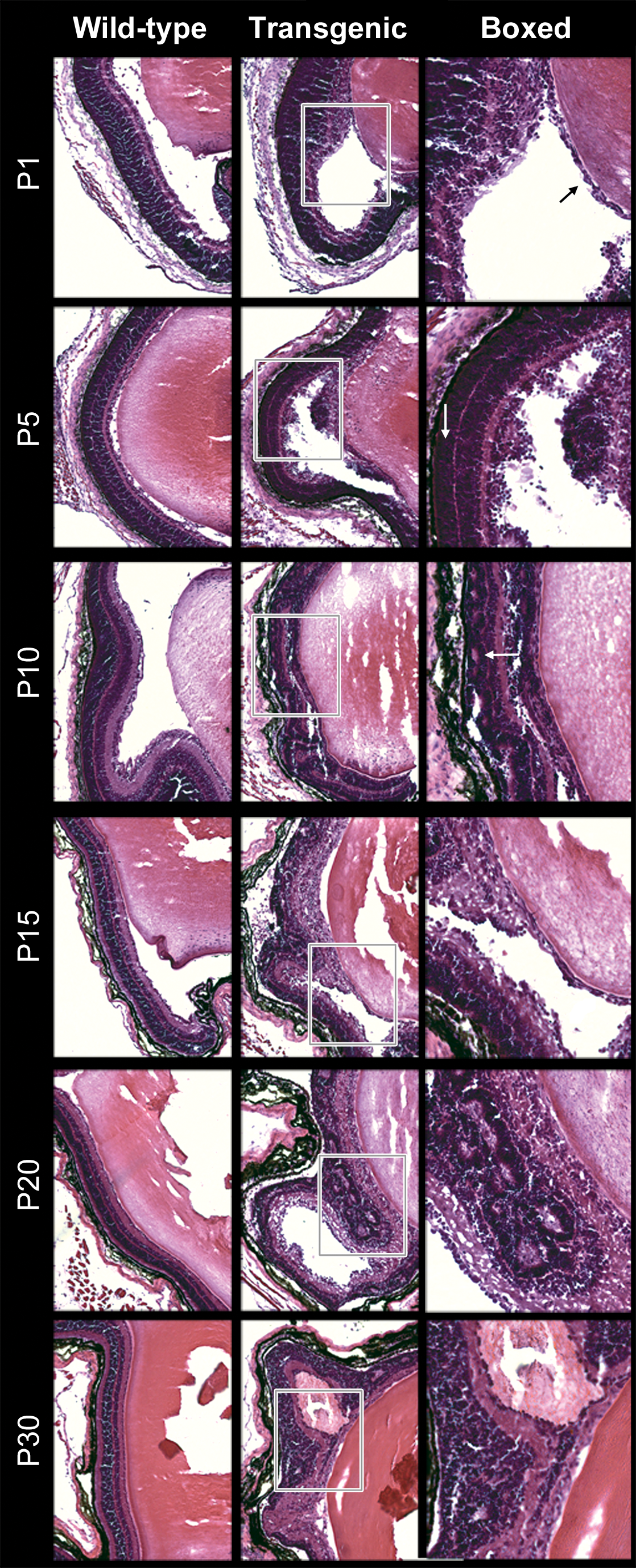

Supplement: Figure S2 — Hematoxylin/eosin staining of wild-type and transgenic retina. Postnatal day 1 (P1), P5, P10, P15, P20 and P30 with magnifications of the boxed areas of transgenic retinas shown on the right. Arrow in P5 points to the ONL where no obvious signs of rosette formation are seen. Arrow in P10 points to rosette formation in the ONL. Boxed region in P15 shows to the retrolental cell mass surrounding the lens (not seen in the wild-type). Boxed regions in P20 and P30 indicate the large-scale distortion of retinal histology possibly caused by tractional forces exerted on the retina. (TIF) [file pone.0042488.s002.tif]

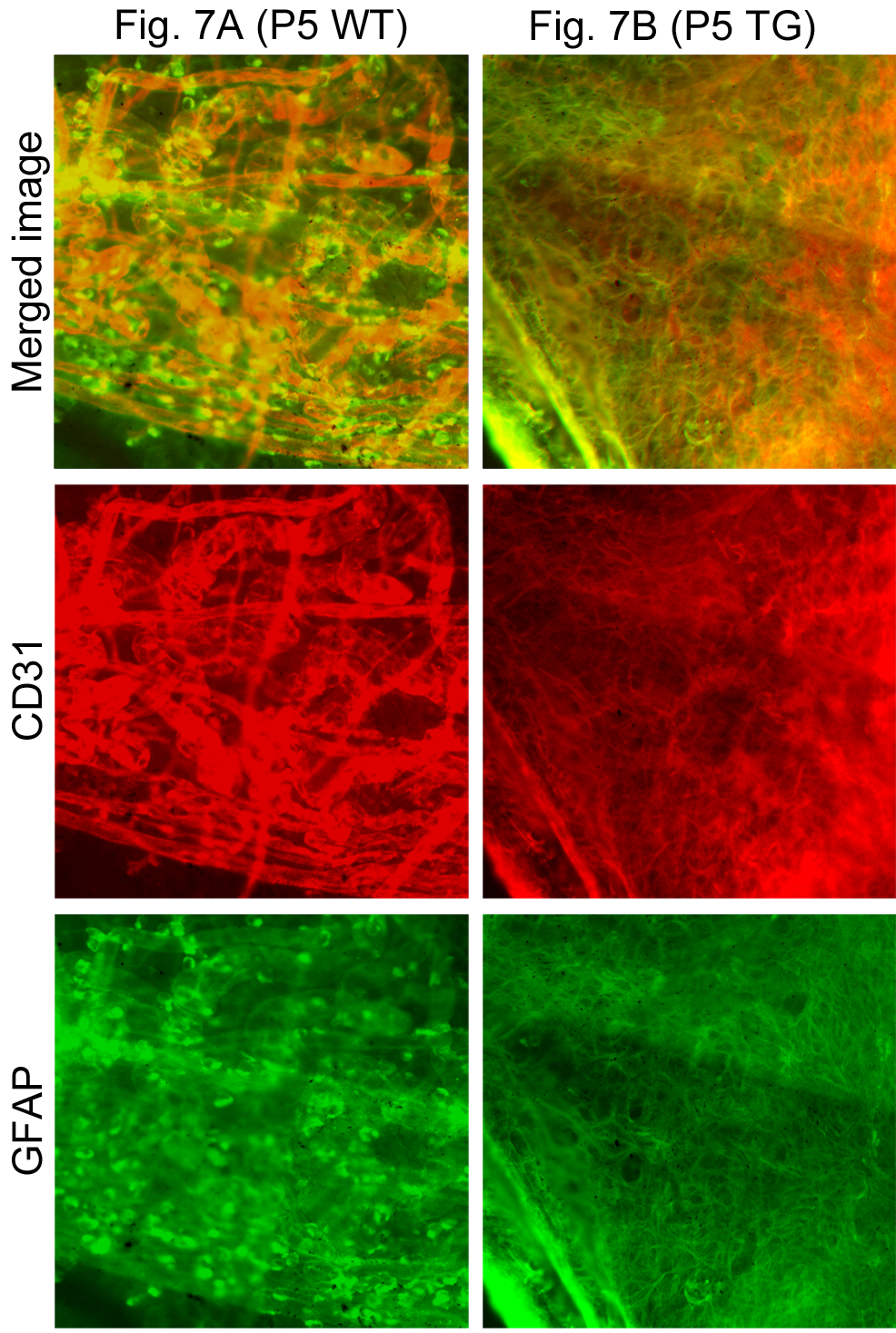

Supplement: Figure S3 — Hyaloid vasculature express CD31 and GFAP. Merged and split two channel images of the boxed regions in Figure 7A–B, shows hyaliod vasculature remnants at P5 at high magnification. Note the denser meshwork of CD31 (red) and GFAP (green) positivity in the transgenic hyaloid as compared to the wild-type. (TIF) [file pone.0042488.s003.tif]

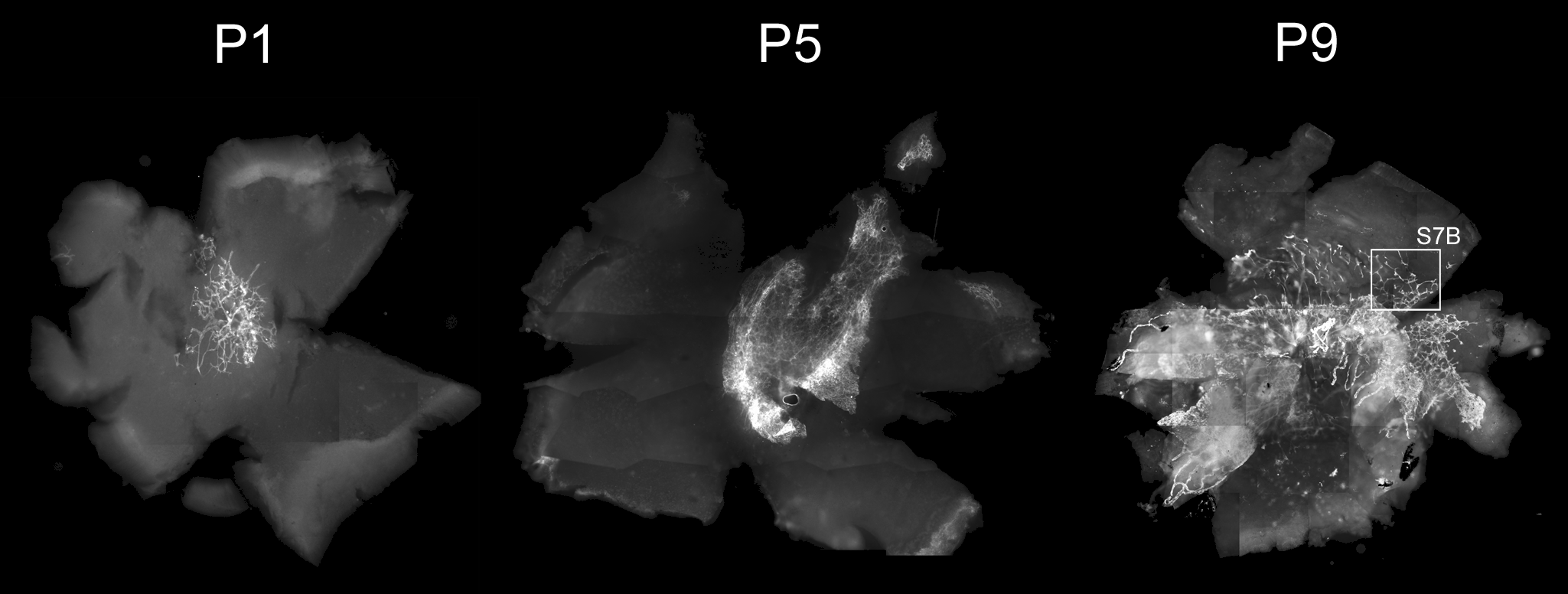

Supplement: Figure S4 — Time-course for blood vessel extension in transgenic retinas. Post-natal day (P) 1, 5 and 9 retinas stained for CD31. At P1, the vasculature emerging from the optic nerve exit forms an irregular mass of vessels. At P5, the mass of irregular vessels have expanded, but without any apparent underlying vascular network. At P9, emerging sprouting vessels (eg. boxed area) starts to vascularize the retina, eventually giving rise to the irregular network of capillaries shown in Fig. 7J (P60) and Figure S5B (P20). The boxed area in P9 depicts the same area shown in Figure S7B. (TIF) [file pone.0042488.s004.tif]

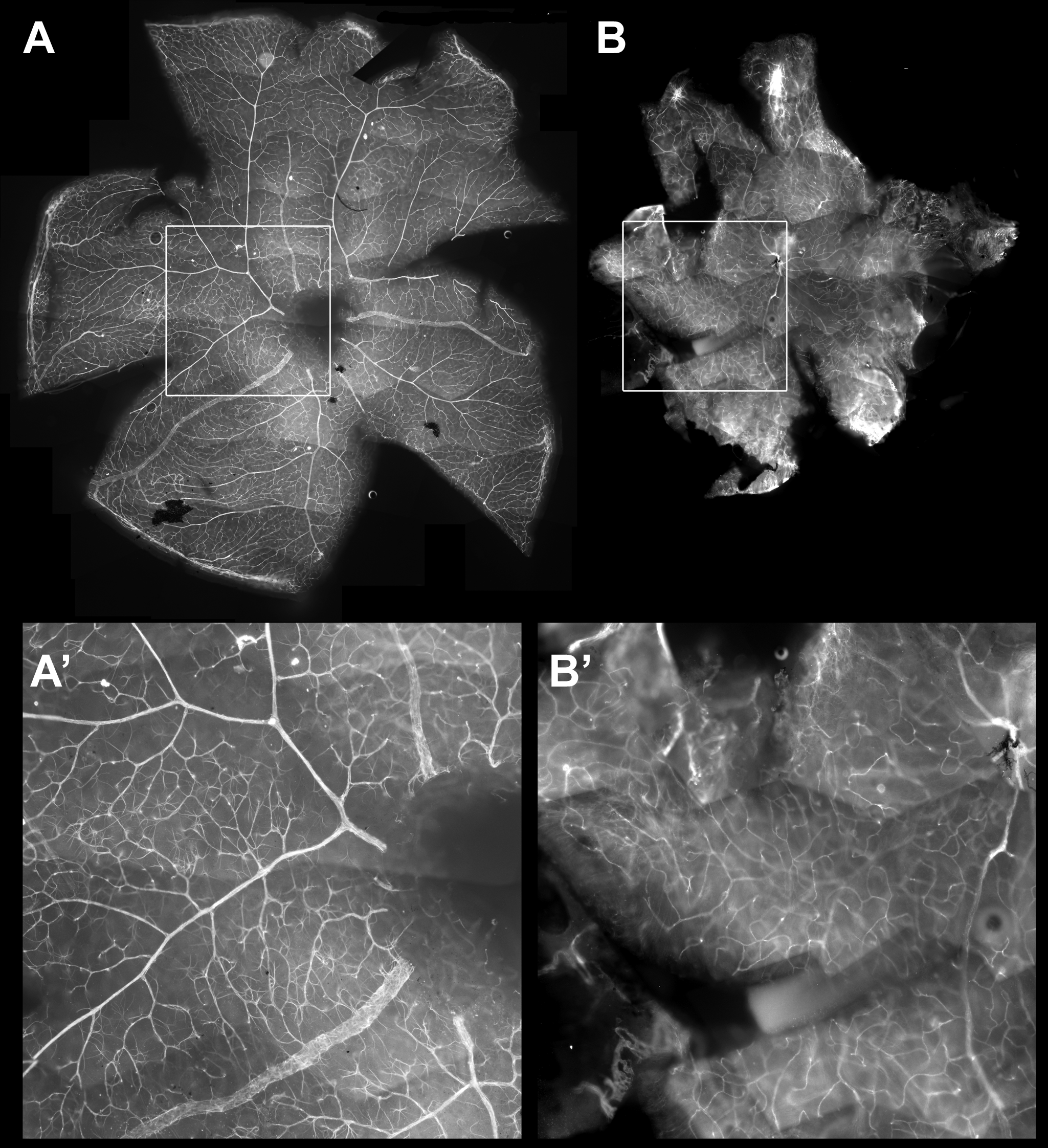

Supplement: Figure S5 — CD31 staining on P20. CD31 staining of P20 wild-type (A, A′) and transgenic (B, B′) retinas reveal the failure of transgenic retinas to form normal trunk vessels. Those that have formed have very few branch points and thus appear to have limited connectivity with the extensive and disorganized capillary network of the transgenic retina. (TIF) [file pone.0042488.s005.tif]

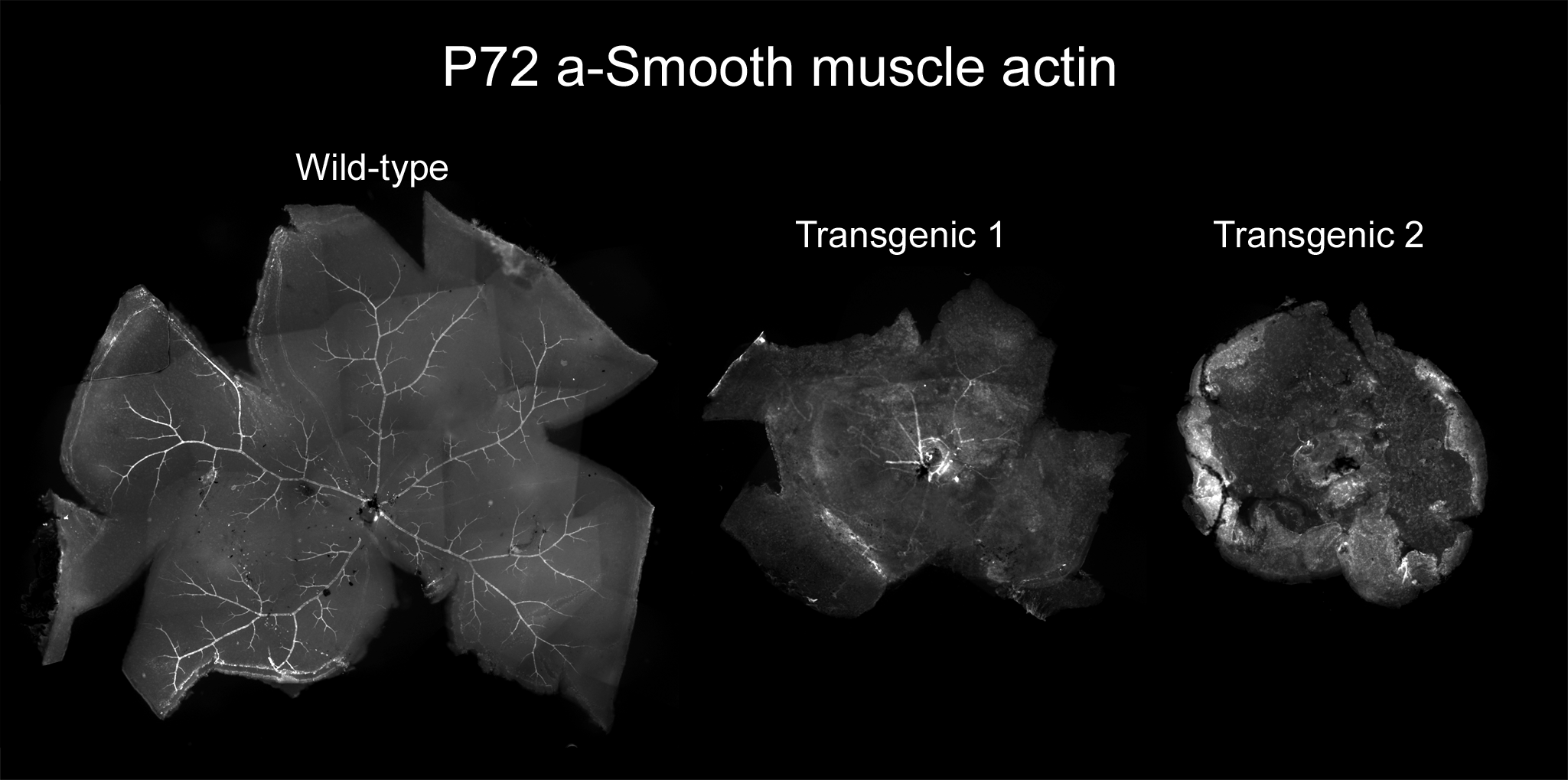

Supplement: Figure S6 — Lack of α-SMA immunoreactivity in transgenic retinas. Staining with an antibody to α-SMA immunoreactivity on flat-mounts of wild-type and transgenic retinas. (TIF) [file pone.0042488.s006.tif]

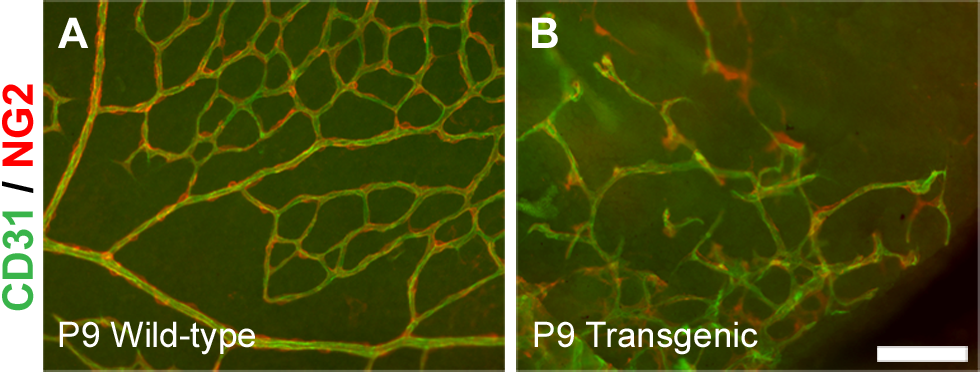

Supplement: Figure S7 — Association of mural cells with vessels in transgenic retinas. Co-labeling of CD31 and NG2 in flat-mounts of wild-type (A) and transgenic (B) retinas on postnatal day 9 (P9) demonstrated that NG2-positive cells remained associated with vessels in the transgenic mice. Scale bar A–B 100 µm. (TIF) [file pone.0042488.s007.tif]

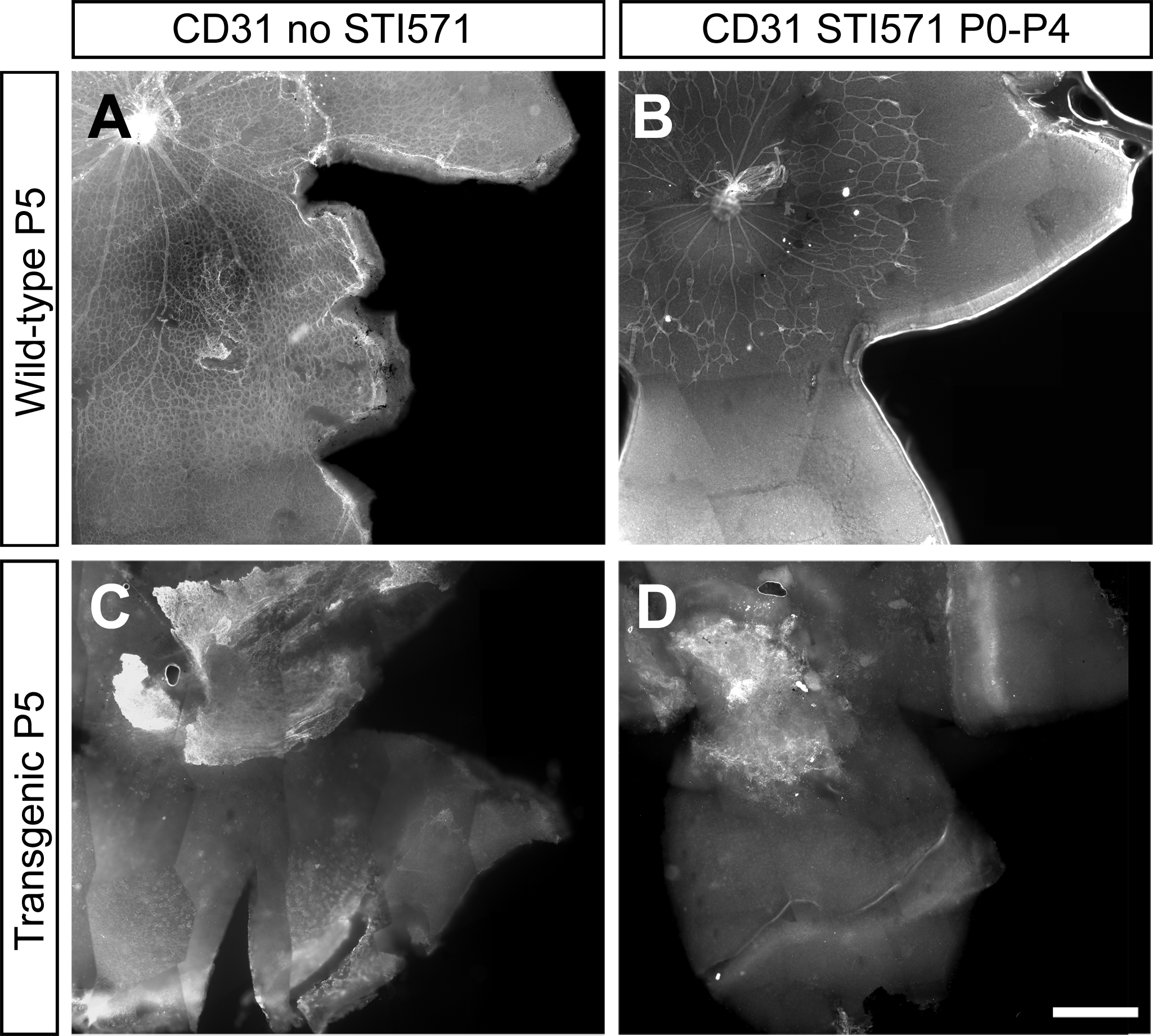

Supplement: Figure S8 — Effect of STI571 treatment on postnatal day 0 (P0) to P4. Suppression of PDGF signaling in wild-type mice by STI571 during the early postnatal period, inhibited vascularization (B). Compare to panel A without treatment. No difference was detected in transgenic mice (C, D) where CD31 stained cells remained as an irregular mass near the optic nerve exit. Scale bar A–D 500 µm. (TIF) [file pone.0042488.s008.tif]

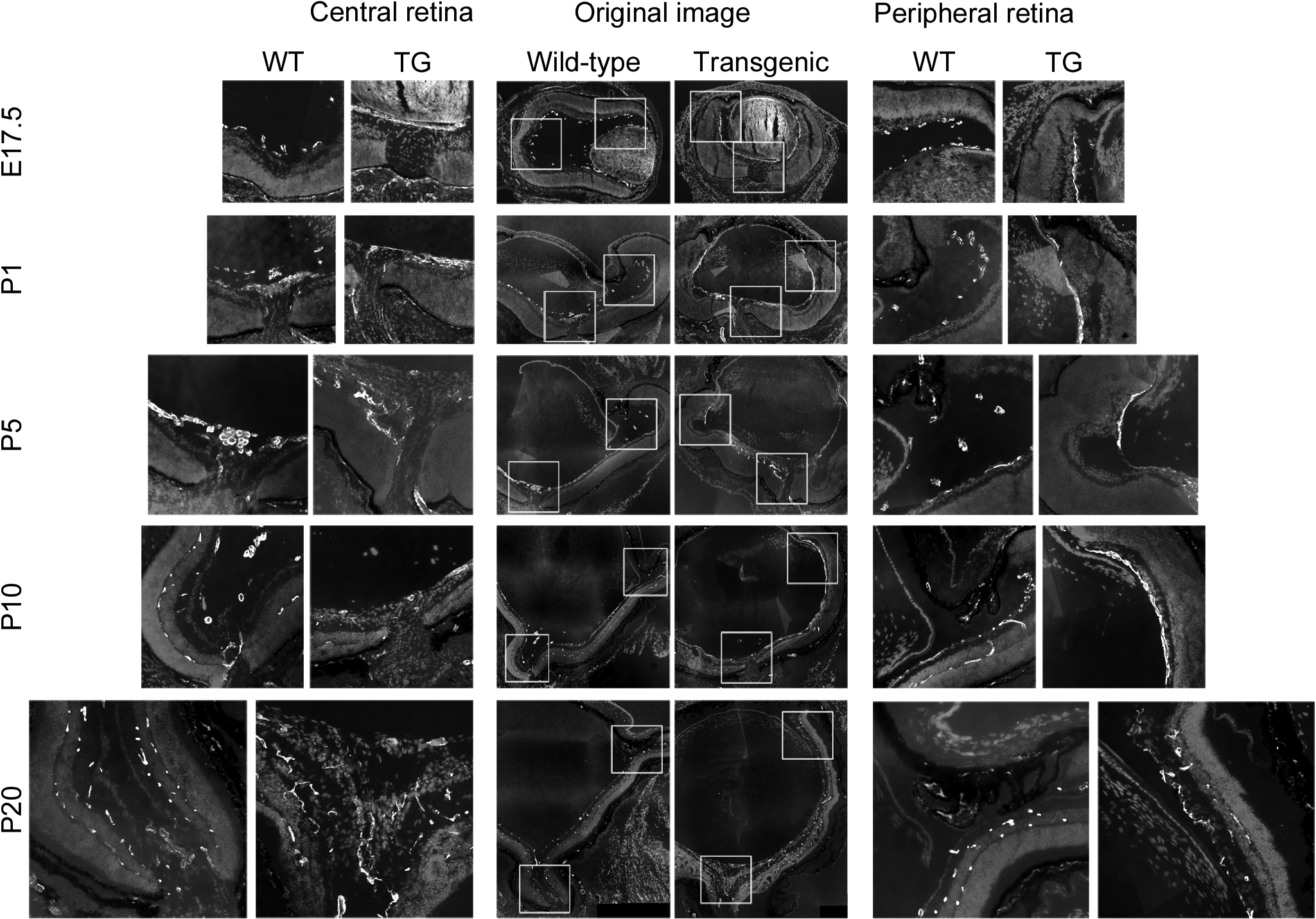

Supplement: Figure S9 — Comparative time series of central- and peripheral parts of wild-type and transgenic retinas. Cross sections of embryonic day (E)17.5 and postnatal day (P) 1, 5, 10, 20 wild-type (WT) and transgenic (TG) retinas stained for NG2. The central two columns depict the low magnification images where magnified regions of central retina (left-most two columns) and peripheral retina (right-most two columns) are indicated by white boxes. (TIF) [file pone.0042488.s009.tif]
