# Supplementary material for: Platelet-Derived Growth Factor Over-Expression in Retinal Progenitors Results in Abnormal Retinal Vessel Formation
Source: PLoS One. 2012 Aug 3;7(8):e42488. doi: 10.1371/journal.pone.0042488 (PMC3411765; doi:10.1371/journal.pone.0042488)
Supplement: Table S1 — List of antibodies used in this study. (DOCX) [file pone.0042488.s010.docx]

**Supplementary Table 1**

List of antibodies used in this study

| **Antibody** | **Origin** | **Dilution** | **Manufacturer** |
| --- | --- | --- | --- |
| Ap2α | mouse | 1:200 | DSHB, 3B5 |
| Calbindin | rabbit | 1:1000 | Chemicon, AB1778 |
| CD31 | rat | 1:500-1000 | Pharmingen, #553370 |
| Cleaved Caspase-3 | Rabbit monoclonal | 1:200 | Cell signaling, 5A1 |
| GFAP | rabbit | 1:500 | Dako, Z0334 |
| GFAP | mouse | 1:800 | Sigma, G3893 |
| Isl1/2 | mouse | 1:200 | DSHB, 40.2D6 |
| Ki67 | rat | 1:100 | DAKO, Clone TEC-3 |
| Lim1/2 | mouse | 1:20 | Developmental studies hybridoma bank (DSHB), 4F2 |
| Lim3 | mouse | 1:200 | DSHB, 67.4E12 |
| Nestin | mouse | 1:100 | DSHB, Rat-401 |
| Neurofilament 150kD | rabbit | 1:1000 | Chemicon, AB1981 |
| Neurofilament 165kD | mouse | 1:200 | DSHB, 2H3 |
| NG2 | rabbit | 1:400 | Millipore, AB5320 |
| Pax2 | rabbit | 1:400 | Covance, PRB-276P |
